# Supplementary material for: Patient costs for prevention of mother-to-child HIV transmission and antiretroviral therapy services in public health facilities in Zimbabwe
Source: PLoS One. 2021 Aug 18;16(8):e0256291. doi: 10.1371/journal.pone.0256291 (PMC8372940; doi:10.1371/journal.pone.0256291)
Supplement: Attachment — Submitted filename: Plos One review comments .docx [file pone.0256291.s002.docx]

Dear Sir,

The authors objectives were to estimate costs associated with seeking and receiving PMCT and ART treatment from a household perspective. This is an interesting paper for programmers and policy makers providing care for people living with HIV and AIDs.

I have some comments the Authors can use to improve the quality of their manuscript.

a) The authors should be explicit about the costing approach used and describe it in more detail. It appears a bottom up or micro costing methodology was used but there is no reference to this
b) What are the implication of using potential earnings to input for lost time? If unemployment is high in Zimbabwe implying not much opportunity costs for the time loss, this would bias the cost estimates upwards. The Authors must critically comment about this. A sensitivity analysis would have been in order to explore uncertainty surrounding such assumptions.
c) Please present summary table of the costs, by the standard cost categories i.e. medical, non-medical and indirect costs etc(Refer to Drummond). Present these separately for PMCT and ART. By comparing the two, you could gain some insight if PMCT cost profile is markedly different from ART profile, which may be useful. For example, PMCT women are generally not very sick and may walk to and from ANC compared to ART patients who are often sick, have others accompanying them and my use different and more costly modes of transport. And in view of this, can author explain why the mean time to care for PMCT and ART are not substantially different, I would expect this given the explanation above.
d) The costs are described as low (page 13) begging the question compared to what?
e) It would be useful to express the annual costs a share of total annual household income or expenditure on health, this would give an idea about burden which can be compared with other available data published in the literature.
f) Limitation section should include re-call biased, as patients are likely to accurately report costs for recent than relatively more distant events or illness episodes. One can do quick robust checks to test this premise and report on findings
g) Selection bias is another limitation, as only individuals seeking care in facilities were interviewed, so results can not be generalised to the general populations. The author can use Heckman models to estimate their costs and control for this bias
