# Supplementary material for: Patient costs for prevention of mother-to-child HIV transmission and antiretroviral therapy services in public health facilities in Zimbabwe
Source: PLoS One. 2021 Aug 18;16(8):e0256291. doi: 10.1371/journal.pone.0256291 (PMC8372940; doi:10.1371/journal.pone.0256291)
Supplement: Attachment — Submitted filename: Responses to Reviewers.docx [file pone.0256291.s003.docx]

ICAP at Columbia University, Zimbabwe Country Office
107 King George
Avondale
Harare
Zimbabwe

Editorial Office

PLOS ONE

**Re: Edits/Response to Reviewers for “Patient costs for prevention of mother-to-child HIV transmission and antiretroviral therapy services in public health facilities in Zimbabwe”**

Dear Editor

We appreciate the opportunity to resubmit our work to your journal. We would like to thank you for helpful comments and suggestions. The manuscript has been modified accordingly, with, revisions in tracked changes. Responses to the reviewers’ suggestions are given below, in bold font.

Editor’s Comment

Please review comments below and address. Please note PLOS ONE data policy. For the third party data not owned by the authors, PLOS ONE requires that the authors provide all information necessary (e.g. URLs, contact information, dataset titles, etc) for other researchers to access the same data used to present the findings of their study and to confirm that they accessed the data in the same manner they expect future researchers to do so, and did not receive special privileges from the third party.  It is not sufficient to note that data is available with the Zimbabwe Ministry of Health. Further, if the data are owned by the authors, PLOS ONE requires the data to be made available unless there are specific legal or ethical considerations for doing so.

***Response: We have uploaded the data as additional supporting information. We have also provided the details of the contact person for any additional clarification i.e Dr Brian Moyo , Epidemiologist at the Zimbabwe Ministry of Health’s AIDS and TB Unit, e-mail-*** [***moyobk1@gmail.com***](mailto:moyobk1@gmail.com)***. Phone +263 774021723..”***

Comment 1. Please ensure that your manuscript meets PLOS ONE's style requirements, including those for file naming. The PLOS ONE style templates can be found at

<https://journals.plos.org/plosone/s/file?id=wjVg/PLOSOne_formatting_sample_main_body.pdf> and

<https://journals.plos.org/plosone/s/file?id=ba62/PLOSOne_formatting_sample_title_authors_affiliations.pdf>

***Response: The manuscript has been revised to match the PLOS ONE’s style requirements.***

Comment 2. Please provide additional details regarding participant consent. In the ethics statement in the Methods and online submission information, please ensure that you have specified how verbal consent was documented and witnessed.

***We have elaborated in the manuscript on page 6-7 on the details of participant consent. Briefly, to obtain informed oral consent, interviewers read the consent form to eligible potential participants in the language that was preferable to the individual and provided them the opportunity to ask any questions. The consent form covered all procedures, potential risks and benefits, and who to contact to report complaints or concerns. After potential participants indicated that they clearly understood the content of the informed consent form, they provided verbal consent by repeating the consenting statement. Those not willing to take part, would read a refusal statement. The interviewer signed an attestation that consent was provided. Those that were enrolled in person were provided a copy of the consent form.***

Comment 3. In your Methods section, please provide additional information about the participant recruitment method and the demographic details of your participants. Please ensure you have provided sufficient details to replicate the analyses such as:

a) the recruitment date range (month and year),

***The recruitment date ranged from September-November 2017 as written in the first sentence on page 6.***

b) a description of any inclusion/exclusion criteria that were applied to participant recruitment,

***The inclusion and exclusion criteria is now reflected on page 6. Briefly, at each site, we selected a cross-sectional convenience sample among adult patients (aged ≥18 years) receiving either outpatient PMTCT services in ANC (pregnant women and postpartum women with HIV) or ART services (ART and pre-ART patients) at the facility on the day of data collection and for whom the date of encounter was not the first visit at the facility. Exclusion criteria used included individuals who were not enrolled in the PMTCT or ART programs at the site, individuals aged less than 18 years of age, individuals who were newly registered PMTCT or ART patients in the health facility to ensure that individuals in the sample have had experience with PMTCT or ART services in the facility, individuals who do not consent to be interviewed in English, Shona, or Ndebele.***

c) a table of relevant demographic details,

***Table 1 on page 9 already includes the demographics details.***

d) a statement as to whether your sample can be considered representative of a larger population,

***We have added in the limitations section on page 16 that “This study sample may not be representative of all PMTCT and ART patients in Zimbabwe”.***

e) a description of how participants were recruited, and

***This is already described in the Facilities and Participants subsections on page 5 and 6.***

f) descriptions of where participants were recruited and where the research took place.

***This is already described in the Facilities and Participants subsections on page 5 and 6.***

Comment 4. Please include additional information regarding the survey or questionnaire used in the study and ensure that you have provided sufficient details that others could replicate the analyses.

For instance, if you developed a questionnaire as part of this study and it is not under a copyright more restrictive than CC-BY, please include a copy, in both the original language and English, as Supporting Information. Moreover, please include more details on how the questionnaire was pre-tested, and whether it was validated.

***The evaluation made use of a standard patient questionnaire developed by CDC, which they used in other countries where they have supported similar studies. It was adapted to the local context and translated to Shona and Ndebele languages.***

Comment 5. We note that you have indicated that data from this study are available upon request. PLOS only allows data to be available upon request if there are legal or ethical restrictions on sharing data publicly. For information on unacceptable data access restrictions, please see <http://journals.plos.org/plosone/s/data-availability#loc-unacceptable-data-access-restrictions>.

***Data have been uploaded as additional supporting information.***

Comment 6. In your revised cover letter, please address the following prompts:

a) If there are ethical or legal restrictions on sharing a de-identified data set, please explain them in detail (e.g., data contain potentially identifying or sensitive patient information) and who has imposed them (e.g., an ethics committee). Please also provide contact information for a data access committee, ethics committee, or other institutional body to which data requests may be sent.

***Upon release of the final report, third-party researchers may e-mail one of the MOHCC PIs listed in the protocol to gain access to the processed data. Permission given to any third party shall be communicated in writing, stating the terms and conditions that need to be observed by the party when using the data, they would have been given access to.***

***Data can be accessed in writing, from the MOHCC Epidemiologist Dr Brian Moyo, who is based at MOHCC AIDS and TB Programme office at 2^nd^ floor Mkwati Building in Harare, via the following e-mail,*** [***moyobk1@gmail.com***](mailto:moyobk1@gmail.com)***. Phone +263 774021723.***

Comment 7. b) If there are no restrictions, please upload the minimal anonymized data set necessary to replicate your study findings as either Supporting Information files or to a stable, public repository and provide us with the relevant URLs, DOIs, or accession numbers. Please see <http://www.bmj.com/content/340/bmj.c181.long> for guidelines on how to de-identify and prepare clinical data for publication. For a list of acceptable repositories, please see <http://journals.plos.org/plosone/s/data-availability#loc-recommended-repositories>.

***Data has been uploaded and Ministry of Health and Child Care will provide data upon request and as mentioned above, we have provided the details in the online submission manager of how data can be accessed. i.e. “Data are available from the MoHCC, as STATA Format named PACE Study Patient Data. Contact Dr Brian Moyo,*** [***moyobk1@gmail.com***](mailto:moyobk1@gmail.com)***. Phone +263 774021723.***

Comment 8. PLOS requires an ORCID iD for the corresponding author in Editorial Manager on papers submitted after December 6th, 2016. Please ensure that you have an ORCID iD and that it is validated in Editorial Manager. To do this, go to ‘Update my Information’ (in the upper left-hand corner of the main menu), and click on the Fetch/Validate link next to the ORCID field. This will take you to the ORCID site and allow you to create a new iD or authenticate a pre-existing iD in Editorial Manager. Please see the following video for instructions on linking an ORCID iD to your Editorial Manager account: <https://www.youtube.com/watch?v=_xcclfuvtxQ>

***The ORCID iD for the first author is*** [***https://orcid.org/0000-0001-6512-2832***](https://orcid.org/0000-0001-6512-2832)***. The paper will be linked to the first author ORCID iD when re-submitting the revised version.***

Comment 9. Please review your reference list to ensure that it is complete and correct. If you have cited papers that have been retracted, please include the rationale for doing so in the manuscript text, or remove these references and replace them with relevant current references. Any changes to the reference list should be mentioned in the rebuttal letter that accompanies your revised manuscript. If you need to cite a retracted article, indicate the article’s retracted status in the References list and also include a citation and full reference for the retraction notice.

***Our reference list have been revised to match the expected standards.***

All authors concur with the re-submission. This work has neither been published nor is it simultaneously being considered for publication elsewhere. Neither I, nor any of my co-authors, have any conflicting interests.

We look forward to hearing from you regarding whether or not these revisions have improved the manuscript such that the revised version can be published in PLOS ONE.

Yours sincerely,

Innocent Chingombe
