# Supplementary material for: Patient costs for prevention of mother-to-child HIV transmission and antiretroviral therapy services in public health facilities in Zimbabwe
Source: PLoS One. 2021 Aug 18;16(8):e0256291. doi: 10.1371/journal.pone.0256291 (PMC8372940; doi:10.1371/journal.pone.0256291)
Supplement: Attachment — Submitted filename: Responses to Reviewers 16 June 2021-1.docx [file pone.0256291.s004.docx]

ICAP at Columbia University, Zimbabwe Country Office
107 King George
Avondale
Harare
Zimbabwe

Editorial Office

PLOS ONE

**Re: Edits/Response to Reviewers for “Patient costs for prevention of mother-to-child HIV transmission and antiretroviral therapy services in public health facilities in Zimbabwe”**

Dear Editor

We appreciate the opportunity to resubmit our work to your journal. We would like to thank you for helpful comments and suggestions. The manuscript has been modified accordingly, with, revisions in tracked changes. We had missed some comments and have now responded to the reviewers’ suggestions as detailed below, in bold italicised font.

**Reviewer #1:**

The authors objectives were to estimate costs associated with seeking and receiving PMCT and ART treatment from a household perspective. This is an interesting paper for programmers and policy makers providing care for people living with HIV and AIDs.

***Thank you***

Comment 1: The authors should be explicit about the costing approach used and describe it in more detail. It appears a bottom up or micro costing methodology was used but there is no reference to this

***We have added the section with a description on the methodology on page 8 of the revised manuscript***

Comment 2: What are the implication of using potential earnings to input for lost time? If unemployment is high in Zimbabwe implying not much opportunity costs for the time loss, this would bias the cost estimates upwards. The Authors must critically comment about this. A sensitivity analysis would have been in order to explore uncertainty surrounding such assumptions.

***We aligned the income based on the information on income sources provided by the participants. We drew the income information from several sources. As part of the sensitivity analysis, we measured minimal wage and maximum casual wage rate. As the economic crises progresses in Zimbabwe; the health system has to advance to accommodate the need to earn money by patients.***

Comment 3: Please present summary table of the costs, by the standard cost categories i.e. medical, non-medical and indirect costs etc(Refer to  Drummond). Present these separately for PMCT and ART. By comparing the two, you could gain some insight if PMCT cost profile is markedly different from ART profile, which may be useful. For example, PMCT women are generally not very sick and may walk to and from ANC compared to ART patients who are often sick, have others accompanying them and my use different and more costly modes of transport. And in view of this, can author explain why the mean time to care for PMCT and ART are not substantially different, I would expect this given the explanation above.

***We have presented these in the table. The cost difference between the groups was very small. With an ART profile-people are being put into treatment earlier especially since we didn’t look at first visits. We also focussed on refill visits to the specific service centres versus outpatient visits in an emergency situation. We did account for such visits but they were not the primary focus and were not reported as frequently.***

Comment 4: The costs are described as low (page 13) begging the question compared to what?

***We have compared to the household monthly income and health sector budget per capita.***

Comment 5: It would be useful to express the annual costs a share of total annual household income or expenditure on health, this would give an idea about burden which can be compared with other available data published in the literature.

Comment 6: Limitation section should include re-call biased, as patients are likely to accurately report costs for recent than relatively more distant events or illness episodes. One can do quick robust checks to test this premise and report on findings

***This has been added***

Comment 7: Selection bias is another limitation, as only individuals seeking care in facilities were interviewed, so results can not be generalised to the general populations. The author can use Heckman models to estimate their costs and control for this bias.

**We tried to obtain information from those who did not attend visits but the sample size was not large enough**

**Reviewer #2:**

This is a nice study with contact with a considerable sample of ART and PMTCT cohort in Zimbabwe.  It demonstrates a cost to individuals to access "free" health care services.

***Thank you***

Comment 1: There are a few areas where perhaps more clarification can be made to outline procedures for those unfamiliar with clinical processes in Zimbabwe.  The site selection process that removed poorly functioning clinics poses some potential bias as clients at those sites likely occur much more cost if the clinic is not run well, and its unclear the size of those poorly functioning clinics to know if they represent a large part of the national cohort, as it represents some percentage of the 565 excluded clinics - 1/3 of all sites in the country.

***More clarifications have been added to the methodology***

Comment 2: If included in the questions to clients, often in addition to wages lost many clients lose employment opportunities due to the recurrent absences they need to incur to attend ART appointments. Were clients asked if they have lost jobs due to recurrent absences?

***We have included further clarifications on these calculations, see page 8***

Comment 3: Additionally the authors discuss client visits with "appointments" versus "without appointment" visits and its unclear if in Zimbabwe these "appointments" are for a certain day or if they are given a time slot within the day which may affect the amount of time spent at the facility. For example, are clients given an appointment on 5 April or 5 April at 1400.

***Appointments are per day and do not drill down to the time***

Comment 4: Further details of the selection process for phone interview will be helpful as clinic staff identified clients eligible for phone interview had to include only those who had phones and it's not clear what percentage of the ART/PMTCT cohort in Zimbabwe has a phone available for followup.  It seems unusual that the authors could not find enough clients who missed even one appointment so perhaps they couldn't find enough who also had a phone?

***This is described on page 7 of the manuscript. Because participants enrolled on-site may visit the facility more frequently, resulting in overestimation of patient costs (since more frequent facility visits entail higher costs), the evaluation also included phone interviews for adult ART or PMTCT patients who had at least one prior appointment at the facility and had missed their most recent appointment***

Comment 5: The authors report, "The data underlying the results presented in the study are available from Zimbabwe Ministry of Health and Child Care."  I am not sure if that qualifies as readily available and defer to the editors.

***We uploaded all the required data***

All authors concur with the re-submission. This work has neither been published nor is it simultaneously being considered for publication elsewhere. Neither I, nor any of my co-authors, have any conflicting interests.

We look forward to hearing from you regarding whether or not these revisions have improved the manuscript such that the revised version can be published in PLOS ONE.

Yours sincerely,

Innocent Chingombe
